# Activity and cellular distribution of ORF3a mutants of SARS-CoV-2 variants of concern

**DOI:** 10.1099/jgv.0.002135

**Published:** 2025-08-06

**Authors:** Ulrike Breitinger, Zeina Ihab Seifeldin Zakaria, Haya Alaa Mahgoub, Anna-Lena Wiessler, Esra Tuerker, Carmen Villmann, Hans-Georg Breitinger

**Affiliations:** 1 Department of Biochemistry, German University in Cairo, New Cairo, Egypt; 2 Institute for Clinical Neurobiology, University Hospital, Julius-Maximilians-University Würzburg, Würzburg, Germany

**Keywords:** activity of ORF3a, cellular distribution, recombinant viral proteins, SARS-CoV-2 variants of concern, viroporin inhibitors, viroporins

## Abstract

Infection with SARS-CoV-2 continues to be a threat to human health. Despite successful immunization campaigns, effective treatment of COVID-19 remains an essential need to help patients and prevent the spread of new virus strains. Viroporins are intracellular ion channels that are essential for virus replication and release, thus presenting promising pharmaceutical targets. Mutations found in variants of concern (VOC) are expected to increase the virulence of the new virus strains. Recognizing the effects of these mutations at the molecular level is essential for the development of improved therapies. Here, we characterized the putative viroporin ORF3a found in VOCs of SARS-CoV-2, using expression constructs containing a myc-tag for identification, and an optional membrane-directing signal peptide. Additionally, constructs containing N-terminal fluorescence protein tags were prepared. Expression and cell surface transport in HEK-293 cells were studied using Western blot and dot blot assays, and the cellular distribution of fluorescent-marked ORF3a was studied using subcellular organelle markers and high-resolution fluorescence microscopy. Viroporin activity of all ORF3a constructs was assessed using cell viability and metabolic assays, as well as patch-clamp recordings of recombinant ORF3a. All ORF3a mutants were expressed well in the recombinant system, and the presence of a signal peptide increased expression on the cellular surface. Intracellular distribution was similar for all variants. The VOC mutants ORF3a-S171L and ORF3a-Q57H showed reduced cytotoxic activity and sensitivity to the viroporin inhibitor rimantadine, respectively, suggesting these positions to be relevant for ORF3a function and a starting point for the search of novel antiviral drugs.

## Introduction

Coronaviruses (CoVs) belong to the order *Nidovirales*, family *Coronaviridae* and subfamily *Coronavirinae* [1]. They have long been known to infect humans, mainly causing mild respiratory infections. Recently, two major outbreaks of respiratory diseases caused by severe acute respiratory syndrome (SARS) CoV were reported, in 2002–2003 (SARS-CoV), and more recently, the worldwide pandemic of COVID-19 in 2019 (SARS-CoV-2). Both outbreaks occurred due to crossover of animal β-CoV to humans [1].

**Fig. 1.**
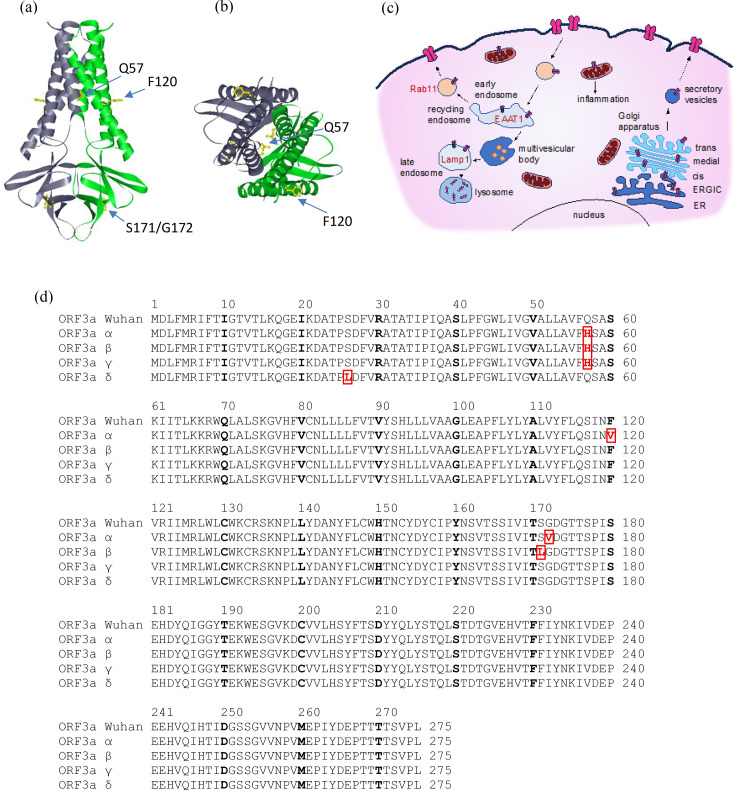
Structure and cellular targets of ORF3a. (a, b) The positions of amino acid exchanges in comparison to 3a-Wuhan of ORF3a are indicated in the structure. Structural data were taken from the cryo-EM structure of SARS-CoV-2 ORF3a in lipid nanodiscs [14]. Structural data are provided from amino acid position 40; therefore, 3A-S26L is not included in the structure. (**a**) Side view. (**b**) Top view. (**c**) Cellular location and trafficking of ORF3a. (**d) **Sequences of ORF3a variants tested in this study (adapted from [3]). Mutations are indicated.

SARS-CoVs are enveloped, positive-sense, single-stranded RNA viruses, containing ~30 kb of RNA arranged into 13-15 ORFs, encoding 29 proteins. Spike (S), envelope (E), membrane (M) and nucleoprotein (N) are the four structural proteins. The S protein binds to the host receptor angiotensin-converting enzyme 2 (ACE2) through the receptor-binding domain in the S1 subunit, while the S2 subunit is responsible for anchoring the S protein in the membrane [2]. The most abundant structural protein in the viral envelope is the M protein, which, together with the N protein, is wrapped around the RNA forming the nucleocapsid [2]. The E protein is found in the virus capsid, although its main function is intracellular. It is mainly expressed in the endoplasmic reticulum (ER) and Golgi membranes of infected cells, acting as a viroporin and intracellular mediator, being involved in virus pathogenesis, assembly and release [2,3]. In addition to the core proteins, CoVs encode several accessory proteins that are thought to contain functions not necessarily required for virus replication but are involved in pathogenicity [4]. SARS-CoV-2 ORF3a is the largest of the accessory proteins, comprising 272 amino acids. Like the E protein, ORF3a is characterized as an integral membrane protein, proposed to act as the second putative viroporin in infected cells and promoting virus release [3], although its function as a viroporin is still under discussion. ORF3a is the largest putative viral ion channel yet identified, containing three transmembrane domains (Fig. 1a, b) with the N-terminus located at the luminal side of the ER and the C-terminal domain directed to the cytosol [3]. ORF3a is located mainly in the Golgi region but also found in the ER, late endosomes, lysosomes [5] and the trans-Golgi network (TGN) (Fig. 1c) [3]. Expression in the plasma membrane (PM) was reported for ORF3a from SARS-CoV-2 [3]. ORF3a activates NLRP3 (Nucleotide-binding oligomerization domain, Leucine-rich Repeat and Pyrin domain containing) inflammasome by inducing secretion of interleukin-1β and nuclear factor NF-κB [6]. The cytoplasmic domain of ORF3a in SARS-CoV was reported to induce pro-apoptotic reactions by activating the mitochondrial p38 MAP kinase pathway [7]. As a defence mechanism against virus entry, ORF3a disturbs the normal process of cellular autophagy through colocalization with lysosomes and interaction with VPS39 of the homotypic fusion and protein sorting (HOPS) complex. This interaction can be blocked by the binding of HOPS with RAB7, which prevents assembly of the fusion machinery, leading to the accumulation of unfused autophagosomes [8].

SARS-CoV-2 continues to evolve, and new variants of the virus have been observed regularly, with some new variants being associated with higher infectivity and/or mortality than previous strains [9,10]. These variants of concern (VOCs) are a particular challenge since they are usually more virulent and may have acquired resistance to existing therapies. Thus, understanding the – potentially modified – pathomechanisms of the novel virus strains is an essential prerequisite for effective therapy against the virus.

Here, we investigated the activity and the cellular distribution of the ORF3a protein of SARS-CoV-2 VOCs, namely, the alpha, beta, gamma, delta and omicron variants. The mutations that were found in these VOCs were Q57H, F120V and G172V (α); Q57H and S171L (β); Q57H (γ); and S26L (δ) (Fig. 1d), while no new ORF3a mutation was detected in the omicron version (Omicron/B1.1.529 BA.1 sub-lineage) at the time of the investigation. Later omicron variations (Omicron/B1.1.529 BA.2 sub-lineage/BA.4 sub-lineage/BA.5 sub-lineage) showed a mutation at the position T223I [9] which was not published at the time of the investigation and is thus not included here. We investigated the localization of recombinantly expressed 3a-Wuhan protein and the VOC mutations to different cellular compartments using fusion constructs of all investigated ORF3a mutants containing N-terminal GFP or red fluorescent protein (RFP) tags. The effect of a membrane-directing signal peptide (SP) on transport to the PM was tested on the WT ORF3a-Wuhan protein (3a-SP). Activity of ORF3a mutants was compared to the wild type in cell viability and metabolic assays, and ion channel activity was studied using patch-clamp electrophysiology. Cellular distribution of all VOC variants was similar to the wild type. Mutants S171L and Q57H showed a reduced cytotoxicity and reduced sensitivity to the viroporin inhibitor rimantadine.

## Methods

### ORF3a mutations

Original recombinant SARS-CoV-2 ORF3a (Wuhan) had been cloned previously into the pRK9 and pRK11 vector using BamHI and HindIII restriction sites [11]. The pRK9-variant contained an N-terminal myc-tag (EQKLISEEDL), corresponding to the C-terminal amino acids (410–419) of human c-myc protein in addition to a membrane-directing signal peptide (MWTPRVPPPRPALSFFLLLLLGVTYG, referred to as SP), taken from the murine semaphorin-6B precursor. The pRK11 construct contained an N-terminal myc-tag for Western blot and dot blot analyses but no SP. Mutations were introduced using either overlap extension PCR or QuikChange mutagenesis. When using QuikChange mutagenesis, we introduced a silent restriction site to identify clones that had taken the mutation. All primers were custom-made by biomers.net (Ulm, Germany).

### Cell culture and transfection

HEK293 cells (ATCC, LGC Standards GmbH, Wesel, Germany) and COS-7 cells (African green monkey kidney cells; CRL-1651; ATCC – Global Bioresource Center, Manassas, VA, USA) were cultured in 10 cm tissue culture Petri dishes in Dulbecco’s Modified Eagle Medium (DMEM; Sigma-Aldrich, Deisenhofen, Germany) supplemented with 10% FBS (Invitrogen, Karlsruhe, Germany) and penicillin/streptomycin (Sigma-Aldrich, Deisenhofen, Germany) at 5% CO_2_ and 37 °C in a water-saturated atmosphere. For electrophysiological experiments, cells were plated on acetone-treated glass coverslips in 24-well plates and transfected 1 day after passage using 1 µg of target cDNA, 1 µg of GFP cDNA and 3 µg of polyethyleneimine (PEI) (Sigma-Aldrich, Deisenhofen, Germany) per well. The transfection mixture was incubated for 20 min before adding dropwise to the cells. Measurements were performed 2–3 days after transfection.

COS-7 cells were transfected using a DEAE-Dextran transfection protocol. To label cellular compartments, plasmid DNA of GFP-tagged ORF3a variants was co-transfected with different compartment markers [dsRed-ER for the ER, mApple-SiT-N-15 (TGN), mCherry-Mito-7 (mitochondrial marker), DsRed-Monomer-Mem (membrane) or Lamp1-mNeonGreen (endosome)]. One microgram of DNA in 30 µl PBS and 10 mg ml^−1^ DEAE-Dextran were mixed and added to the cells. After an incubation of 30 min at 37 °C, cells were washed, and 12 µM chloroquine in 2 ml culture medium was added to the cells. The medium was exchanged again after 2 h, and cells were used for immunocytochemical stainings 48–72 h after transfection.

### Membrane preparation and Western blotting

HEK293 cells were grown in 6 cm plates, transfected 24 h after passage (compare Cell Culture and Transfection) and incubated for 72 h to ensure protein expression. Cells were harvested with cold PBS (1.5 mM KH_2_PO_4_, 6.5 mM Na_2_HPO_4_, 3.0 mM KCl, 137 mM NaCl) and centrifuged at 2,000 ***g***, 4 °C. The pellet was resuspended in hypotonic buffer [20 mM K-phosphate, pH 7.4, 5 mM EDTA, 5 mM EGTA, 1 tablet complete^™^ Mini, Protease Inhibitor Cocktail (Sigma-Aldrich, Deisenhofen, Germany)] and kept on ice for 20 min. Cell homogenization was performed in a cell homogenizer, and cells were centrifuged at 17,000 r.p.m. for 20 min, 4 °C. Pellets were resuspended in hypotonic buffer, and protein concentration was determined in preparation for SDS-PAGE. Twenty micrograms of protein in SDS loading dye (2× loading dye: 0.125 M Tris-HCl, pH 6.8, 20% glycerol, 4% SDS, 0.02% bromophenol blue, 0.2 M DTT) were loaded per lane and subjected to SDS-PAGE and Western blotting. We used a combination of first antibody c-myc-tag mouse antibody (Abcam, USA) and secondary antibody AP-conjugated goat-anti-mouse IgG (Carl Roth, Karlsruhe, Germany). The blot was visualized using 0.03% nitro blue tetrazolium (NBT) and 0.02% 5-bromo-4-chloro-3-indolyl-phosphate (BCIP) (both Carl Roth, Karlsruhe, Germany) in substrate buffer (100 mM Tris-HCl, pH 9.5; 100 mM NaCl; 5 mM MgCl_2_).

### Dot blot analysis

HEK293 cells were grown in 6 cm plates and transfected. Three days after transfection, cells were washed with PBS. Anti c-myc mouse antibody (Abcam, USA) in full medium was applied onto the living cells and incubated at 37 °C for 2 h. Cells were washed three times in PBS, before adding secondary AP-conjugated anti-mouse antibody (Carl Roth, Karlsruhe, Germany) in full medium for 1 h. Cells were again washed three times in PBS, harvested, and protein concentration determined. Thirty micrograms of protein per dot were applied in quadruplicates directly onto the dot blot membrane. The blot was developed using BCIP and NBT in substrate buffer and imaged. Pixel intensity was quantified using ImageJ software without further correction of the raw data. Pixel intensities for each well were normalized to the total pixel intensity of all peaks of each treatment.

### Immunocytochemical staining

Transfected HEK293 or COS-7 cells with ORF3a variants and compartmental markers were fixed using 4% paraformaldehyde with 4% sucrose in PBS for 20 min at room temperature (RT, ~21 °C). Fixed cells were washed three times with PBS followed by blocking and permeabilization using 5% goat serum with 0.2% Triton X-100 in PBS for 30 min. When no cellular compartment marker was transfected, primary antibodies against ERGIC-53 (ALX-804-602 C100, RRID:AB_2051363, Enzo Biochem, Farmingdale, NY, USA), cis-Golgi/GM130 (610822, RRID:AB_398141, BD Transduction Laboratories, Heidelberg, Germany) or Rab11 (715300, Thermo Fisher, Karlsruhe, Germany) were used in a dilution of 1 : 250 in PBS containing 5% normal goat serum and incubated for 1 h at RT. Incubation of secondary antibodies goat-anti-rabbit-Cy3 (111-165-003, RRID:AB_2338000, Dianova, Hamburg, Germany) and goat-anti-mouse-Cy3 (115-165-003, Dianova, Hamburg, Germany), diluted 1 : 500 in PBS containing 5% goat serum, was performed for 1 h in the dark. Cell nuclei were stained with 4′,6-diamidino-2-phenylindole in PBS for 5 min, and cells were mounted on a microscope slide in Mowiol.

### Confocal microscopy and image acquisition

Images were captured using an Olympus Fluoview ix1000 microscope using an UPLSAPO 60× oil objective and diode lasers of 405, 495 and 550 nm in 1,024×1,024 pixels. Fiji/ImageJ software was used for image processing [12].

### Cell viability assay

One hundred and twenty microlitres of a HEK293 cell suspension were seeded to give ~20,000 cells per well of a 96-well plate. Cells were transfected 1 day after seeding using 0.3 µg of ORF3a or the empty pRK5 vector as a control and 0.6 µg of PEI and applied after 20 min incubation. If needed, 4.5 µl of 30× inhibitor stock (rimantadine in DMEM) was added to achieve a final concentration of 20 µM. Four days after transfection, the MTT assay was performed as described before [13]. The absorbance of formazan was taken in a Victor-3 plate reader (PerkinElmer, Berlin, Germany) at a wavelength of 595 nm. Formation of formazan requires active mitochondria and measures the number of viable cells. Viability of cells transfected with ORF3 cloned into the pRK vector was compared to cells transfected with the control pRK vector. Further controls included GFP-transfected cells to verify efficient transfection, and 200 mM KCl was added to untransfected cells to induce complete cell death. To be able to combine experiments, absorbance readings were divided by control readings (control pRK vector). A minimum of two experiments and four wells per experiment was averaged per mutation.

### Metabolic activity test

Transfected HEK293 cells with different ORF3 variants were prepared in duplicates. Six micrograms of plasmid DNA of ORF3a protein variants or GFP DNA as a control were mixed with 0.1× TE buffer, 2.5M CaCl_2_ and 2× HBS buffer (50 mM HEPES, 12 mM glucose, 10 mM KCl, 280 mM NaCl, 1.5 mM Na_2_HPO_4_) and incubated for 20 min at RT. The prepared transfection reagent was added dropwise to the cells and incubated at 37 °C. The medium was exchanged after 6 h, and fresh medium was added. The cells were used for experiments 48 h after transfection. The cells were detached and counted, and from each sample, 1,000 cells/well were seeded into 96-well plates (white, flat-bottom, Corning 3917, NY, USA). Fifty microlitres of culture media were added over cells. Real-Time Glo^™^ MT Cell Viability (G9712 Promega, Madison, USA) Reagent (enzyme+substrate) was prepared by following the manufacturer’s protocol. Fifty microlitres of the reagent were added to the cells to give a total volume of 100 µl. Luminescence readings were made 3 days after transfection. The luminescence measurements were done at 1,000 ms integration time using the TECAN Spark instrument (Männedorf, Switzerland). For the calibration curve (data not shown), HEK293 cells were seeded into a 96-well plate with 200, 400, 800, 1,600, 3,200 and 6,400 cells and incubated with the assay reagent.

### Electrophysiological recordings and data analysis

HEK293 cells were plated on glass cover slides and co-transfected with GFP for visualizing transfected cells. For the investigation of the current–voltage ramp, we used symmetrical external and internal electrophysiology buffer. External buffer was (in mM) 135 NaCl, 5.5 KCl, 2.0 CaCl_2_, 1.0 MgCl_2_ and 10 HEPES (pH adjusted to 7.2 with NaOH); internal buffer contained (in mM) 140 CsCl, 1.0 CaCl_2_, 2.0 MgCl_2_, 5.0 EGTA and 10 HEPES (pH adjusted to 7.2 with CsOH). Control cells were transfected with the pRK5 vector co-transfected with GFP and compared to cells transfected with ORF3a co-transfected with GFP. The applied voltage ramp ranged from −60 mV to +50 mV in 10 mV steps. For each ORF3a protein, a minimum of six cells were averaged. Average current values at −60 mV were compared and plotted.

## Results

We introduced five mutations originating from SARS-CoV-2 ORF3a VOCs into the pRK vector to investigate their activity in comparison to the original ORF3a from SARS-CoV-2 (Wuhan, China). Fig. 1 highlights the position of the respective ORF3a mutations (Fig. 1a, b), and the cellular compartments which have been described for the cellular distribution of ORF3a (Fig. 1c). The sequence of the WT (Wuhan) and VOC variants of this study is shown in Fig. 1(d). The structural data were taken from the cryo-EM structure of SARS-CoV-2 ORF3a in lipid nanodiscs [14]. The mutation 3A-S26L is not included in Fig. 1a, b, since the available cryo-EM structure does not resolve amino acids up to position 40.

**Fig. 2.**
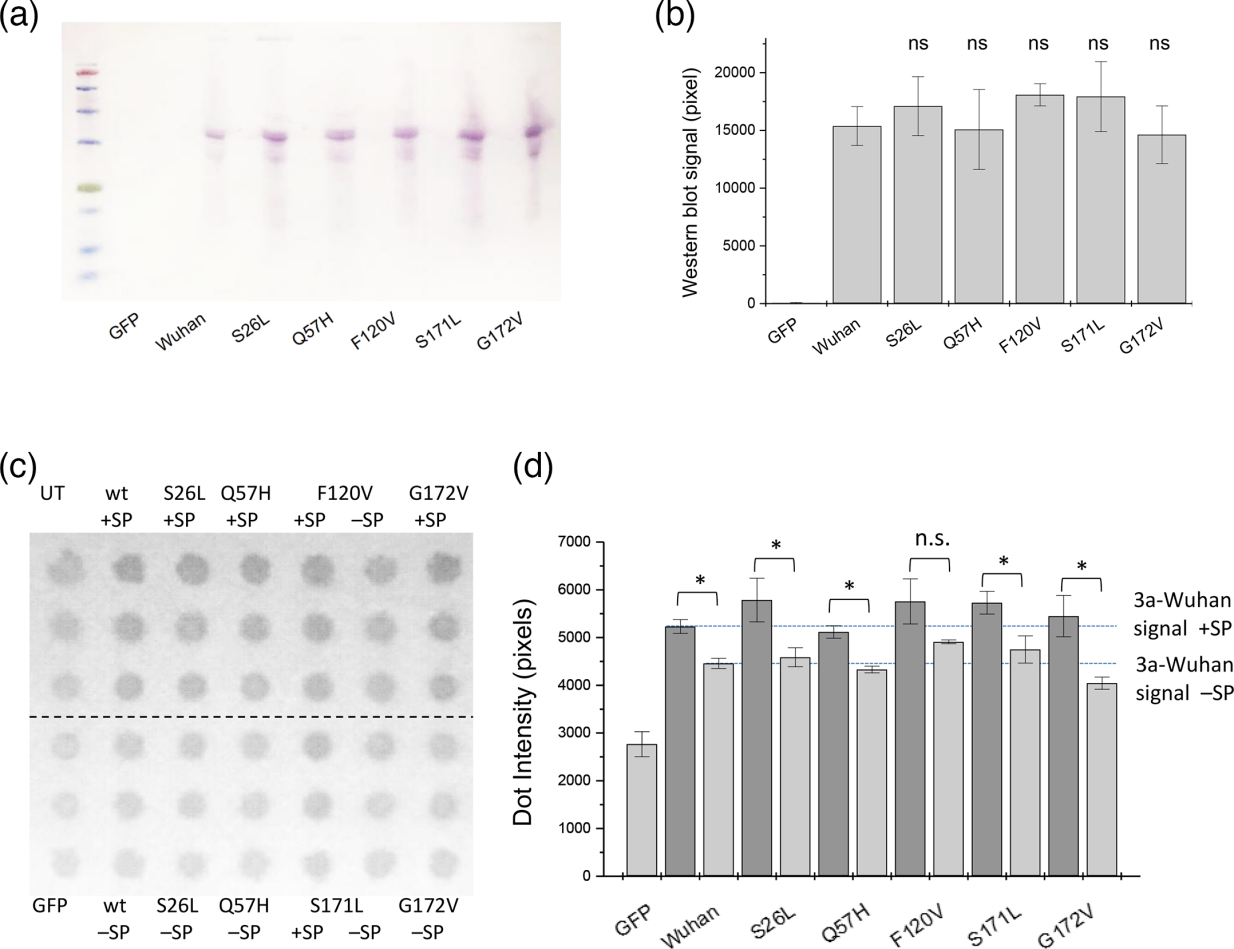
ORF3a recombinant expression. Western blot and dot blot analyses were performed using c-myc mouse first antibody in combination with secondary AP-conjugated anti-mouse antibody. Western blot and dot blot analyses were done on transfected HEK293 cells, expressing 3a-Wuhan strain and VOCs S26L, Q57H, F120V, S171L and G172V. (**a**) Gel image of Western blot analysis for constructs including an N-terminal myc tag. (**b) **ImageJ quantification of two independent Western blot analyses. (**c) **Dot blot analysis of cell surface receptors. HEK293 cells were transfected with the indicated ORF3a mutants, living cells treated with primary antibody, then subjected to dot blot analysis. For dot blot analysis, constructs containing an N-terminal myc-tag and a membrane-directing signal peptide were compared to ORF3a constructs having only an N-terminal myc-tag. (**d) **Triplicate determinations of two independent preparations were analysed using ImageJ. While differences between surface expression of ORF3a with and without signal peptide were statistically significant (except for F120V), differences between ORF3a-Wuhan and mutant proteins were not statistically significant for both variants.

The ORF3a original protein (3a-Wuhan) was cloned into two different vectors: pRK-SP-myc (3a-SP) – including a PM-directing signal peptide plus myc tag, and pRK-myc (3a-myc) – including the myc tag only. Recombinant expression of 3a-myc constructs in HEK293 cells was verified by membrane preparation and Western blotting after transfection applying 20 µg of total protein per lane (Fig. 2a). Signal intensities were compared using ImageJ, averaging signals from two independent experiments and normalizing to the 3a-Wuhan (wt) signal (Fig. 2b). We only found small, non-significant differences in signal intensity and concluded that all constructs expressed ORF3a protein in similar amounts.

To investigate the cell surface expression of ORF3a, HEK293 cells were transfected with ORF3a constructs, live cells were then treated with antibodies and harvested, total protein concentration was determined and 30 µg per dot was loaded in a dot blot apparatus. This procedure ensured that only protein on the outer surface of the cells was detected. Signal intensities from triplicate measurements (Fig. 2c) of two independent assays were compared using ImageJ and compared to a negative control (GFP-transfected cells). Surface expression was significantly higher for the SP-containing constructs, except for 3a-F120V (Fig. 2d). No difference in surface expression was observed between the wild type and all tested ORF3a mutants (Fig. 2d).

**Fig. 3.**
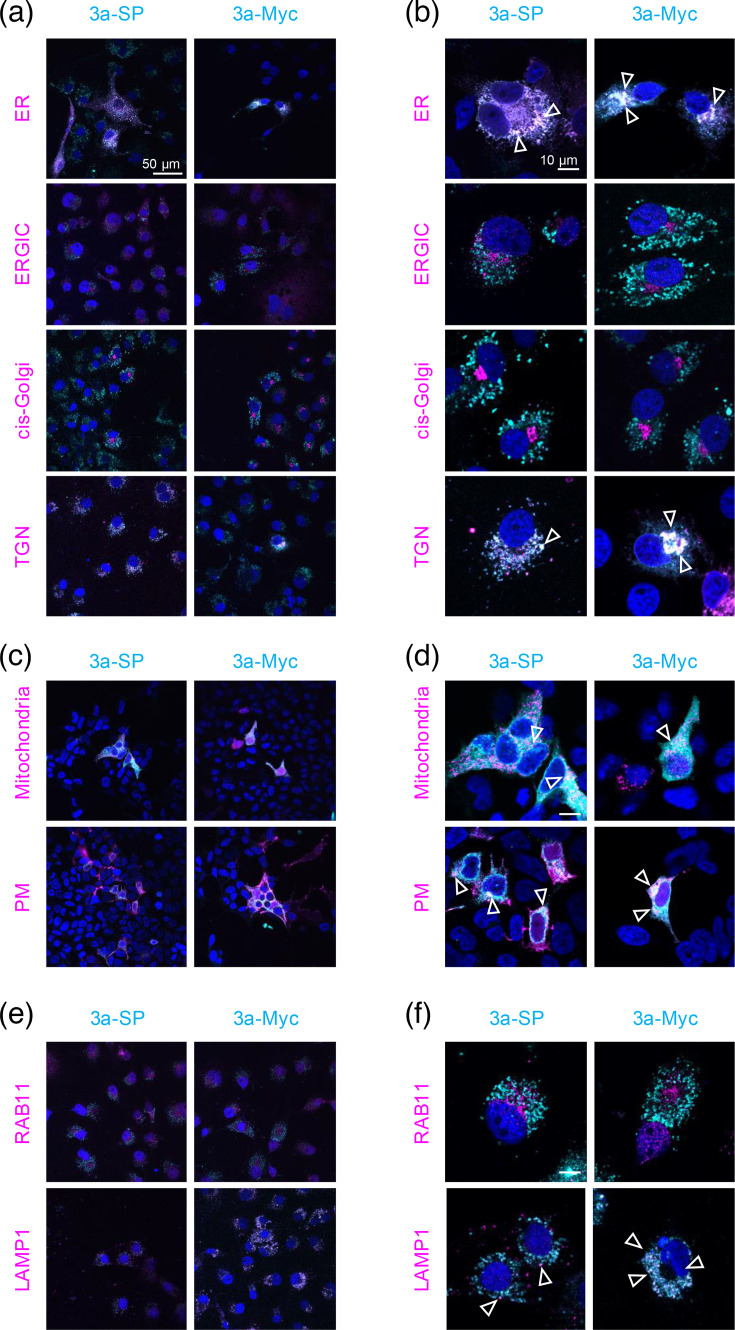
Trafficking of ORF3a with and without a signal peptide. (a) Overview images from COS-7 cell stainings to visualize the cellular compartments ER, ERGIC, cis (cis-Golgi) and trans-Golgi (TGN) (pink) and ORF3a Wuhan strain with (3a-SP) or without (3a-myc) (cyan). (**b) **Enlargements of single representative cells shown in (a). (**c) **Overview images from ORF3a (cyan) co-transfected with plasmids labelling the mitochondria or the PM (pink) of transfected HEK293 cells. (**d) **Enlargements of single representative cells shown in (c). (**e) **Overview images of stainings from the lysosome (LAMP1) and the recycling endosome (Rab11) marker together with ORF3a variants. (**f) **Enlargements of single representative cells shown in (e). In all images, compartments are coloured in magenta and ORF3a proteins in cyan. The subcellular experiments have been performed in three independent biological replicates (*n*=3). The scale bar refers to 50 µm in the overview images (**a, c, e**) and 10 µm in images with single cells (**b, d, f**).

The distribution over different cell compartments (Fig. 1c) of the 3a-Wuhan constructs with either the membrane-directing signal peptide (3a-SP) or a myc-tag (3a-myc) was studied by subcellular compartment analysis via immunofluorescence (Fig. 3), using transfected COS7 cells and HEK293 cells.

Both ORF3a-Wuhan constructs – with and without signal peptide – distributed similarly. Colocalization was observed for ER and the TGN, while only minor signals were detectable in the ER–Golgi intermediate compartment (ERGIC) and the cis-Golgi, most likely due to fast transport of both ORF3a proteins through these compartments (Fig. 3a, b). In the recombinant system, both 3a constructs were seen in mitochondria and at the cellular membrane, arguing that the signal peptide may promote membrane localization without being an essential pre-requisite (Fig. 3c, d). Downstream of membrane insertion, endosomal localization was clearly observed, while ORF3a-SP and ORF3a-myc were never identified in recycling endosomes (Fig. 3e, f).

**Fig. 4.**
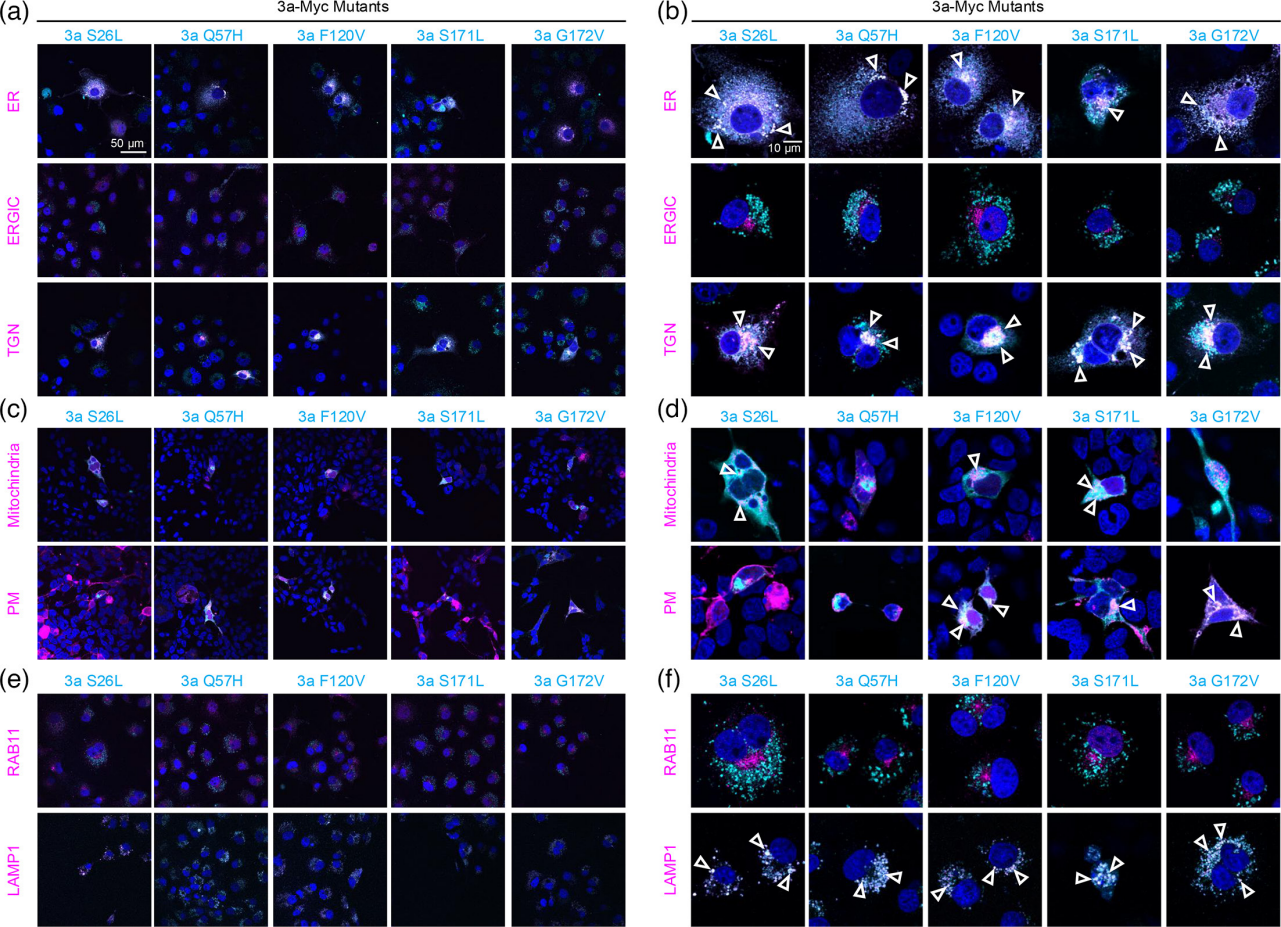
Trafficking of ORF3a VOCs through cellular compartments. Overview images from stainings of COS-7 cells to visualize the cellular compartments ER, ERGIC and Golgi (TGN) shown in (**a)**; compartment markers for mitochondria or PM in transfected HEK-293 cells in (**c);** and the lysosomal (LAMP1) and recycling endosome marker (Rab11) in (**e)**. All images were also stained for the co-transfected ORF3a mutants. (**b) **Single representative cells from images in (a). (**d)** Single cells labelled with compartment markers for mitochondria or PM together with ORF3a variants from images in (c). (**f) **Representative images of single cells transfected COS-7 cells with marker for lysosomes (LAMP1) and early endosomes (Rab11), enlargements from (e). Compartments are coloured in magenta, and ORF3a proteins in cyan. Open arrowheads point to colocalization of compartment marker together with the ORF3a variants. The subcellular localization experiments have been performed in three independent biological replicates (*n*=3). The scale bar refers to 50 µm in the overview images (**a, c, e**) and 10 µm in images with single cells (**b, d, f**).

We then studied the ORF3a mutants from VOC and compared these to the ORF3a-Wuhan construct. ORF3a proteins were N-terminally tagged either with GFP or RFP, which allowed easy localization. The observed compartmental pattern was very similar to the ORF3a-Wuhan. All ORF3a variants ORF3a S26L, Q57H, F120V, S171L and G172V were found to be intensively expressed in the ER and TGN but almost not in ERGIC, again arguing for fast transport of ORF3a variants through ER compartments (Fig. 4a, b). No PM localization was seen for variants S26L and Q57H, while all other variants were detectable at the cell surface; notably, membrane localization of the S171L mutant was low compared to variants F120V and G172V (Fig. 4c, d). The mutations investigated in the ORF3a did not change the intensive colocalization to mitochondria and late endosomes (Lamp1), while no signal was detectable colocalizing with recycling endosomes (Rab11), similar to the observation with the WT constructs (Fig. 4c–f).

**Fig. 5.**
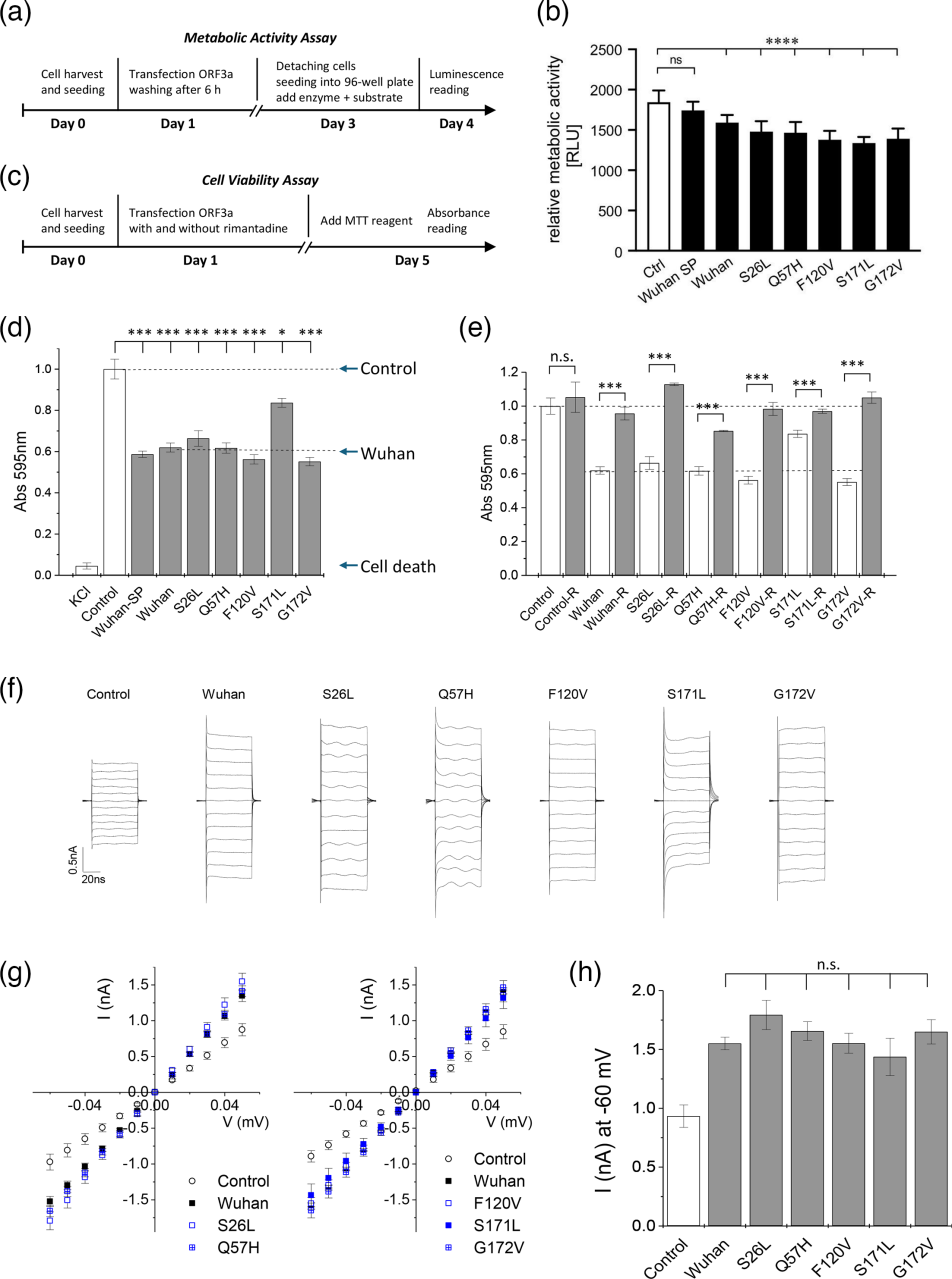
Cell viability, metabolic activity and putative channel activity of ORF3a variants. (a–e) Cell viability assays. (**a) **Schematic timeline of the metabolic activity test. (**b) **Metabolic activities of ORF3a variants were investigated 3 days post-transfection and compared to mock (CTRL)-transfected cells. The activity is shown as relative luminescence values (RLU). Note that 3a-Wuhan and all 3a variants exhibited significantly reduced metabolic activity. Number of experiments: *N*=3, each experiment with six independent data points (*n*=18). (**c) **Schematic timeline for the MTT cell viability test. HEK293 cells were transfected with 3a-Wuhan and 3a-VOC mutations. After 4 days of treatment, cell viability was tested using an MTT assay. (**d) **Control (empty vector) was compared to 3a-SP and 3a-myc and its various VOC mutations (all without SP). Complete cell death was observed after treatment with 200 mM of KCl. All VOC mutations showed significantly reduced cell viability. Cell viability was less reduced after 3a-S171L transfection as compared to 3a-Wuhan expression. (**e) **Rimantadine (20 µM end concentration) was used as a specific viroporin inhibitor to verify specific 3a activity. White column: no inhibitor; black column: 20 µM rimantadine. (**f–h) **Patch clamp electrophysiology on HEK293 cells transfected with empty vector (control) or 3a-Wuhan. (**f) **Whole-cell current traces from HEK293 cells transfected with the indicated ORF3a constructs. Current–voltage ramps were taken between −60 and +50 mV. All cells transfected with 3a-Wuhan and 3a-VOF protein showed increased currents compared to cells transfected with the empty vector. (**g) **Current–voltage traces in the range of −60 to +50 mV. Averages±sem from six to eight cells are shown. (**h) **Current at −60 mV of ORF3a-expressing HEK293 cells versus GFP-expressing control cells. Errors are given as sem. Significance values: *P<0.05; **P<0.01; ***P<0.001, ****P<0.0001.

Involvement of ORF3a in inducing pro-apoptotic reactions has been described in several studies [15,17]. The cell viability assay was established as an activity test for viroporins of the hepatitis C p7 channel and the E protein of SARS-CoV-2 [13,18]. Notably, cell viability may be affected by viral proteins other than viroporins. To test the cytotoxic effect of recombinant ORF3a on transfected cells, we performed two independent tests of cell viability. First, we measured cellular metabolic activity after expression of ORF3a by performing the Real-Time Glo^™^ MT Cell Viability test which uses a substrate that can only be reduced by living cells, giving a luminescence signal which is proportional to the number of active cells. We found significantly reduced cellular metabolic activity for cells transfected with ORF3a constructs after 3 days in culture (Fig. 5a, b). The metabolic assay includes a replating step where dead or weak cells are removed, and the viability of the remaining culture is measured. The test thus shows the effect of viral protein expression on the entire cell culture.

As a second, independent test, we used an MTT-based assay, showing the effect of ORF3a expression on the viability of transfected HEK293 cells. Different from the metabolic activity test, the MTT assay avoids the second splitting step, giving a direct measure of the fraction of cells destroyed by the viral protein (Fig. 5c). Thus, the metabolic activity is the activity of the remaining living cells, transfected or untransfected. In contrast, the MTT test, directly performed at 4 days post-transfection, includes all dead and living cells. This mixture may account for the more pronounced effect on cellular activity compared to the metabolic assay and more reliably represent the consequences of ORF3a toxicity on cellular viability (Fig. 5c, d). To this end, we seeded HEK293 cells in 96-well plates, transfected 1 day post-seeding and added inhibitors to mock-, control- and ORF3a-transfected cells (Fig. 5c). Active inhibitors should suppress ORF3a activity and thereby rescue cell viability. We used 3a-myc constructs (without SP) for all constructs studied in this experiment. Additionally, we compared the activity of Wuhan 3a-SP and 3a-myc to investigate the effect of the additional membrane-directing signal peptide on cell viability. Both Wuhan ORF3a proteins showed the same activity (Fig. 5d), which was significantly reduced compared to the GFP-transfected control. Thus, ORF3a was able to reduce cell viability to an extent similar to that of other bona fide viroporins, this effect being independent of the absence or presence of a signal peptide. All VOC mutants showed similar activity to the 3a-Wuhan wild type, with the exception of 3a-S171L. Cytotoxicity of the 3a-S171L variant was significantly higher than for the GFP control, but significantly less than that of the other ORF3a constructs (Fig. 5d). For all constructs, application of the viroporin inhibitor rimantadine (20 µM) returned cell viability to control levels (Fig. 5e), confirming that reduction of cell viability was indeed due to activity of ORF3a.

The putative ion channel activity of ORF3a constructs in HEK293 cells was tested using SP-containing constructs. To this end, we transfected cells 1 day after seeding and recorded transmembrane currents after an additional 2 days of expression. Individual cells were attached to a recording pipette in the whole-cell configuration, and current responses to a voltage ramp protocol were recorded. Voltage was ramped from −60 mV to +50 mV in 10 mV intervals and whole-cell currents recorded (Fig. 5f). Currents recorded from cells transfected with ORF3a constructs were slightly increased compared to control (mock-transfected) cells, showing a steeper current–voltage relationship compared to untransfected control cells (Fig. 5f, g). This is compatible with a channel activity of surface-expressed ORF3a, as observed with other viroporins. Notably, indirect signalling of intracellular ORF3a, or effects on membrane conductance, cannot be excluded. We observed that all ORF3a constructs increased whole-cell current responses, with the 3a-S171L variant showing the smallest signal, in agreement with its reduced activity in the cell viability test (Fig. 5h).

## Discussion

Throughout the short time of the COVID-19 pandemic, SARS-CoV-2 has shown a high rate of mutation, rapidly generating numerous new strains, several of which were characterized by high infectivity and a rapid distribution among the population. These strains, termed VOCs, differ in infectivity, transmissibility, severity of disease, drug sensitivity and efficacy of neutralization by monoclonal antibodies, convalescent sera or vaccines [9]. In addition, VOC-associated mutations may cause molecular diagnostic tests to lose sensitivity, thereby leading to a delayed diagnosis, increased spread and delayed treatment [9]. VOCs have been reported from various parts of the world, and the most frequent variants include B.1.1.7 (alpha), B.1.351 (beta), B.1.617 (delta), P.1 (gamma) and B.1.1.529 (omicron). All described VOCs showed higher transmissibility than the original Wuhan strain and most pre-existing lineages. Severity of disease varied between the different VOCs, with omicron and beta being less severe than delta and gamma variants [9]. Most of the mutations found in VOCs are located in the S protein. While transmissibility most likely depends on the S protein which is responsible for binding the ACE2 receptor of the host, severity of associated COVID-19 can be affected by several viral proteins, including the ORF3a protein.

In this study, we characterized ORF3a mutations from VOCs of SARS-CoV-2 to evaluate possible differences between the Wuhan strain and VOC proteins. Cellular expression as shown by Western blotting was not different between 3a-Wuhan and VOC mutants, indicating that all variants were normally expressed in the recombinant system. Surface expression was also unaltered for all mutations (Fig. 2).

Several studies have investigated the cellular distribution of the ORF3a protein. Most information was obtained from immunofluorescence data done on recombinantly expressed protein or infected cells. ORF3a was found in PM [19], early endosome [20], late endosome [21,22], lysosomes [5,8], as well as in the ER [23], and Golgi compartments [24]. The C-terminal cytoplasmic domain of ORF3a contains the motif YxxΦ (where x is any amino acid; Φ is an amino acid with a bulky hydrophobic side chain) upstream of an ExD (di-acidic) motif [19]. In general, the di-acidic motif mediates export from the ER [25], whereas the YxxΦ motif directs protein localization to various intracellular compartments [26]. In addition, most YxxΦ motifs are capable of mediating rapid internalization from the PM into endosomes [26]. The juxtaposition of the two different sorting motifs has been found in the cytoplasmic tails of a number of PM proteins [27], suggesting that this may be important for the transport of proteins to the PM [19]. Recently, a novel diG (deletion of the double glycine) motif was described in ORF3a. Similar to the YxxΦ motif, the diG region (aa 187–188) led to ORF3a retention in the Golgi apparatus [28].

In the present study, we investigated the cellular distribution of 3a-Wuhan and 3a-mutations from VOCs in several cellular organelles [ER, ERGIC, cis- and/or TGN, mitochondria, PM, lysosome (Lamp1) and recycling endosomes (Rab11)] to investigate ORF3a protein distribution in detail (Table 1).

**Table 1.** Colocalization of ORF3a protein variants with cellular organelle markers

	Cell line	3a-Wuhan plus SP	3a-Wuhan no SP	S26L	Q57H	T120V	S171L	G122V
PM	HEK	+*+*	++	+	+	++	++	++
Mitochondria	HEK	*++*	++	++	++	++	++	++
Cis-Golgi	Cos7	+	+	nd	nd	nd	nd	nd
TGN	Cos7	+	++	++	++	++	++	++
Lysosomes	Cos7	+	++	++	++	++	++	++
ER	Cos7	+*+*	++	++	++	++	++	++
ERGIC	Cos7	+	+	+	+	+	+	+
recycling endosomes	Cos7	no	no	no	no	no	no	no

+, minor colocalization; ++, intensive colocalization; nd, not determined; no, no colocalization.

Firstly, we investigated the effect of an additional membrane-directing signal peptide on cell surface localization and cellular distribution of ORF3a, generating the construct 3a-SP (including signal peptide from mouse semaphorin 6a) and 3a-myc, containing the myc-tag only. We observed colocalization with the PM for both variants. Notably, the addition of the signal peptide in 3a-SP expression reduced trafficking to TGN, and lysosomes, to a lesser extent to mitochondria, while localization to ER and ERGIC was unaffected. We found 3a-myc-Wuhan and all VOC mutations in the PM, mitochondria, TGN, lysosomes, ER and ERGIC. However, no colocalization was detected for Rab11 which specifically associates with recycling endosomes returning internalized molecules back to the PM.

ORF3a activity was assessed using cellular viability assays in HEK293 cells verified by specific rimantadine inhibition using mock (GFP)-transfected cells as a control. ORF3a expression resulted in significantly reduced viability for the Wuhan strain and all VOC strains. However, when comparing 3a-Wuhan activity to the VOC mutations in the same assay, we found a reduced cytotoxic activity of the 3a-S171L mutant compared to the other VOC-derived constructs. Indeed, a recent *in silico* study investigating 173 residues of the ORF3a protein found 12 mutations that severely affected ORF3a stability. However, only three of these mutations (Y160H, D210Y and S171L) were suggested to cause alterations in secondary structure and protein disorder of ORF3a. Structure analysis and modelling predicted that a turn structure in S171L should be replaced by a coiled coil [29]. Such a structural rearrangement would agree with the altered activity of 3a-S171L found in this study. Furthermore, ORF3a residues Ser171 and Trp193 have been shown to be critical for promoting lysosomal exocytosis and blocking autophagy. Indeed, this pathway is absent in SARS-CoV, which has a glutamate at position 171 [16]. Possibly, the mutation 3a-S171L reduces or abolishes autophagy. However, in the presently investigated cellular distribution, we did not observe any differences in cellular trafficking between 3a-Wuhan and 3a-S171L. Also, the metabolic assay showed no difference in the metabolic activity of 3a-S171L compared to the 3a-Wuhan protein. This discrepancy may be due to reduced mitochondrial activity (measured by the MTT viability assay) in viroporin-damaged cells, while the metabolic activity of these cells may still be high. The position ORF3a-S171L may thus be critical for the activity of ORF3a.

We found that ORF3a activity was sensitive to the channel blocker rimantadine. In the presence of rimantadine, cell viability returned to control levels for all mutants except ORF3a-Q57H. Activity of the ORF3a-Q57H variant was partially but not completely inhibited by rimantadine (*P*=0.034). Residue Gln57 is located in the putative ion channel pore (Fig. 1). A molecular dynamics analysis on several fundamental clades of SARS-CoV-2 suggests that the exchange ORF3a-Q57H constricts the viroporin channel by enabling an inter-transmembrane-domain interaction between Cys81 and His57 [30]. A further modelling study on SARS-CoV-2 proteins revealed dramatic changes in protein structure (RMSD ≥5.0 Å) for Q57H in ORF3a [31]. Blockage of ORF3a and other viroporin channels by amantadine, a close structural relative to rimantadine, has been reported [32]. In our experiments, we observed similar cytotoxic activity of 3a-Wuhan and the 3a-Q57H construct. Still, the proposed Cys81–His57 interaction, or other changes in the protein structure, could interfere with rimantadine binding. Position Q57 appears to be relevant for virus replication, since it was reported that 3a-Q57H was associated with poor replication in Vero-CCL81 cells, but not in bone marrow-derived endothelial progenitor (BEpC) cells [33]. Thus, position ORF3a-Q57H may be relevant for ORF3a function and sensitivity to inhibitors.

Whole-cell patch-clamp electrophysiology was performed to assess the function of ORF3a constructs as viral ion channels. Cytotoxic activity of viroporins as determined by cell viability assays is generally in agreement with membrane channel activity recorded using patch-clamp electrophysiology [13,18], as was also observed in this study. We found increased transmembrane currents for all ORF3a constructs, with the 3a-S171L mutant showing the smallest current signal and the lowest activity in the cell viability assay.

Overall, we found that all mutants of ORF3a in recent VOCs of SARS-CoV-2 showed activity in cellular and patch-clamp assays that were compatible with those of a viroporin. Cellular distribution revealed slight differences between the mutants showing that ORF3a indeed interacts with numerous partners in infected host cells, yet the activity of all mutants is similar to the wild type. Notably, two positions, Q57 and S171, were identified as critical for ORF3a function and distribution, in agreement with reports in the literature that also identified these residues to be relevant for ORF3a folding and activity. The ORF3a protein is an important player in the life cycle of SARS-CoV-2, and its mechanism of action and role in virus propagation require further study.

## Abbreviations

ACE2: angiotensin-converting enzyme 2
BCIP: 5-bromo-4-chloro-3-indolyl-phosphate
CoVs: coronaviruses
DMEM: Dulbecco’s Modified Eagle Medium
E: envelope
ER: endoplasmic reticulum
ERGIC: ER–Golgi intermediate compartment
HOPS: homotypic fusion and protein sorting
M: membrane
N: nucleoprotein
NBT: nitro blue tetrazolium
PEI: polyethyleneimine
PM: plasma membrane
RFP: red fluorescent protein
S: spike
SARS: severe acute respiratory syndrome
SP: signal peptide
VOCs: Variants of Concern
VOCs: variants of concern
